# Increasing Salt Rejection of Polybenzimidazole Nanofiltration Membranes via the Addition of Immobilized and Aligned Aquaporins

**DOI:** 10.3390/pr7020076

**Published:** 2019-02-03

**Authors:** Priyesh Wagh, Xinyi Zhang, Ryan Blood, Peter M. Kekenes-Huskey, Prasangi Rajapaksha, Yinan Wei, Isabel C. Escobar

**Affiliations:** 1 Chemical and Materials Engineering Department, University of Kentucky, Lexington, KY 40506, USA; 2 Department of Chemistry, University of Kentucky, Lexington, KY 40506, USA

**Keywords:** aquaporins, nanofiltration, immobilization, biomimetic

## Abstract

Aquaporins are water channel proteins in cell membrane, highly specific for water molecules while restricting the passage of contaminants and small molecules, such as urea and boric acid. Cysteine functional groups were installed on aquaporin Z for covalent attachment to the polymer membrane matrix so that the proteins could be immobilized to the membranes and aligned in the direction of the flow. Depth profiling using x-ray photoelectron spectrometer (XPS) analysis showed the presence of functional groups corresponding to aquaporin Z modified with cysteine (Aqp-SH). Aqp-SH modified membranes showed a higher salt rejection as compared to unmodified membranes. For 2 M NaCl and CaCl_2_ solutions, the rejection obtained from Aqp-SH membranes was 49.3 ± 7.5% and 59.1 ± 5.1%. On the other hand, the rejections obtained for 2 M NaCl and CaCl_2_ solutions from unmodified membranes were 0.8 ± 0.4% and 1.3 ± 0.2% respectively. Furthermore, Aqp-SH membranes did not show a significant decrease in salt rejection with increasing feed concentrations, as was observed with other membranes. Through simulation studies, it was determined that there was approximately 24% capping of membrane pores by dispersed aquaporins.

## 1. Introduction

A growing research area in water purification is the incorporation of transmembrane water channel proteins, known as aquaporins in the synthetic membranes owing to the excellent permeability and selectivity of aquaporins (aqp) towards water molecules [1–6]. These membranes are called biomimetic membranes because they mimic the function of aquaporins present in lipid bilayer within cell membranes. In recent years, a number of approaches have been adapted from biological concepts and principles to develop biomimetic membranes [2,6–27]. However, there are still many challenges associated with aquaporins based membranes. Generally, aquaporin-based biomimetic membranes developed to date consist of three building blocks: aquaporins, amphiphilic molecules in which the aquaporins are embedded in order to simulate the environment of the lipid bilayer in the cell membranes, and a polymer support structure [22]. These amphiphilic molecules in which the aquaporins are incorporated can be either lipids or polymers. Due to the superior mechanical and chemical properties, block copolymers and amphiphilic polymers have been predominantly investigated for the development of aquaporin-based membranes [2,6,19,22,28–37]. Studies using lipids as the amphiphilic molecules to support aquaporins have shown that these systems were able to maintain membrane integrity [7,12–14,18,23,38,39].

**Figure 1.**
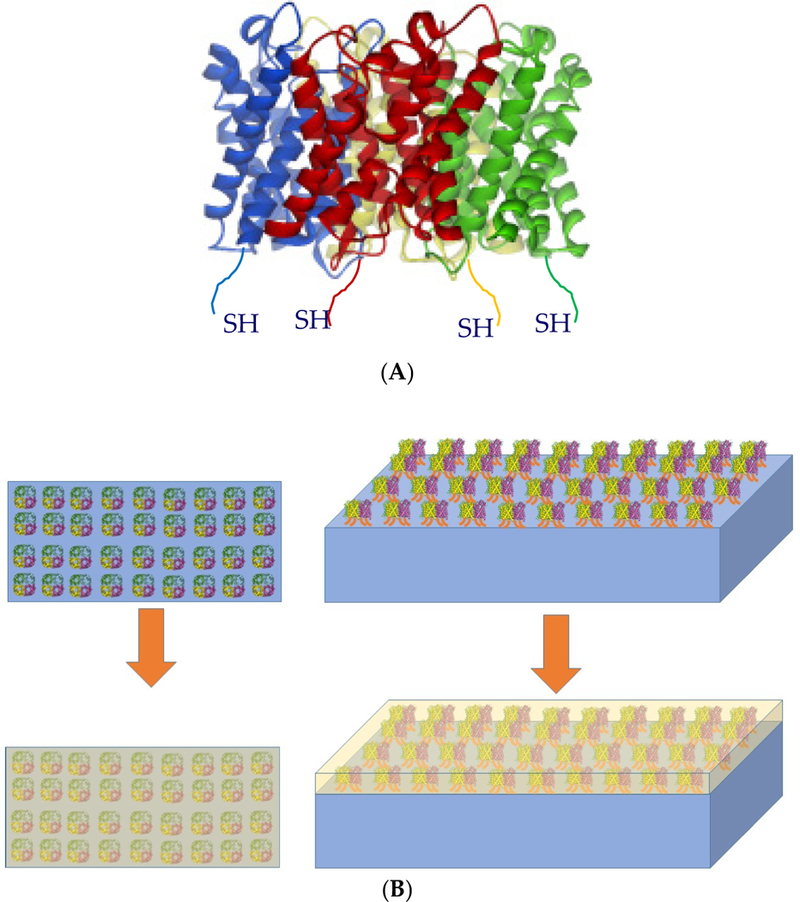
(**A**) Cys modified aquaporin molecule (Aqp-SH). (**B**) Chemical attachment of Aqp-SH on -COOH modfied Polybenzimidazole (PBI-COOH) membrane and in-situ addition of a layer of Polyvinyl alcohol carrying long; alkyl chains (PVA-alkyl).

The widespread application of aquaporin-based membranes faces several challenges with respect to synthesis, s tability and function of the membrane assembly. One of the challenges is to design and prepare biomimetic membranes with embedded and aligned aquaporin proteins without losing their integrity and perfoemance, while providing an additional solid support that is sufficiently porous [23]. Toward this goal_,_ aquaporin censtructs were modified to bear affinity tags or unique amino acids at the N-terminus of the aquaporin molecule, which was used to facilitate directional immobilization. Each aquaporin monomer was modified with a unique amino acid Cys gnoup at the N-torminus right after the first Met (Figure 1A)_,_ and due to the aquaporin tetrameric nature, these Cys group became four anchors for attachment. There are two intrinsic Cys groups in the sequence of aeuaporin. Studies have shown that they are not chemically reactive toward modifieations due to their limited accessibility [4]. Therefore, the engineered Cys would be the only site in aquaporin reactive loward the thiol-specific modificat!on. The presonce of these four Cys anchors per aquaporin tetramer was used to ensure that all tetramers were attached on the membrane surface in alignment with the feed direction.

The objective of this study was to covalently attach Cys modified aquaporins (Aqp-SH) to a polymeric membrane backbone in order to align them in the direction of flow. Polyvinyl alcohol carrying long alkyl chains (PVA-alkyl) was used to bind the remaining sites present on the backbone and to seal the gaps in between attached aquaporin molecules. PVA-alkyl has been previously usrd to enhance the mechanical strength of the membrane assembly and simulate the natural environment for attached aligned aquaporins [40]. Figure 1B provides a schematic of the attachment of Cys modified aquaporins to the membrane backbone.

## 2. Experimental

### 2.1. Materials

#### 2.1.1. Polybenzimidazole (PBI)

Polybenzimidazole (PBI, PBI Performance Products, Inc, Charlotte, INC, USA) was used as the backbone of membrane assembly. PBI has found applications in ion-exchange membranes for fuel cells becauoe of its excellent mechanical, thermal and chemical stability over a wide range of pH [41–43]. The specific polymer composition used in those membranes is poly [2,2’-(1,3-phenylene)-5,5’-bibenzimidazole]. The absence of aliphatic groups and stability of beczimidazole group in PBI area responsible for its applications in a wide range of pH [44]. Hydrogen bonds can tie formed Intramolecularly or intermolecularly due to the heterocycle imidazole ring presented in the repeating unit of PBI molecules [45]. PBI was dissolved in N,N-Dimethylacetamide (Sigma Aldrich, St. Louis, MO, USA) to prepare dope solution.

#### 2.1.2. PVA-Alkyl

PVA-alkyl is amphiphilic in nature with the high hydrophilicity of PVA (Sigma Aldrich, St. Louis, MO, USA) and hydrophobicity of the long alkyl side chains. Being amphiphilic in nature, it can be an excellent synthetic alternative for the lipid bilayer in cell membrane where aquaporins are constituted naturally [22]. PVA-alkyl spontaneously attaches to cell surface, anchoring through hydrophobic interactions between the alkyl chains and the lipid bilayer of the cell membrane without reducing cell viability [46]. It carries 28 alkyl side chains per molecule, and interacts strongly with the lipid bilayer in cell membrane because the alkyl chains anchor to the cell surface at multiple points [47]. Because of these hydrophobic interactions and the tendency of the polymer to protect the cells, PVA-alkyl is proposed to be an excellent material to support aquaporins. Materials required in order to synthesize PVA-alkyl, and the polymer PBI were of the same source and grade that were used in previous studies [40].

#### 2.1.3. AquaporinZ Modification with Single Cysteine at the N-Terminus

**Figure 2.**
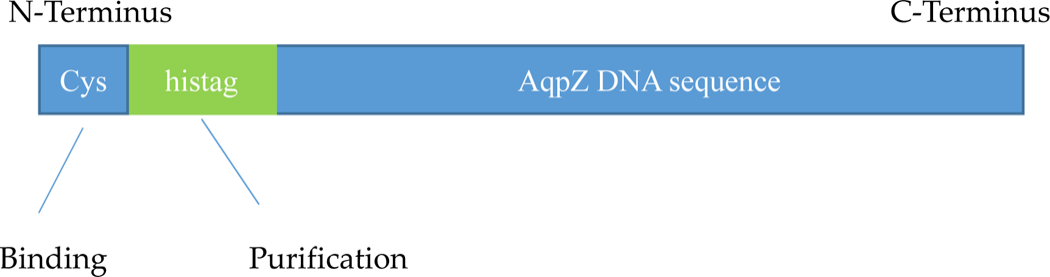
Schematic of Cysteine attachment at the N-terminus of aquaporins using site-directed mutagenesis. Cysteine groups were added to act as anchors in order to attach Aqp on PBI membrane surface.

Cysteine containc a thiol group in its side chain, which ean be used for immobilization. To prevent cysteine from being buried m the structure of AqpZ with limited accessibility for blinding, a cysteine was added before the hit-tag which was used to facilitate protein purification via the conventional metal-affinity chromatograph as shown in Figure 2. In this study, cysteine group was added using QuikChange site-directed mutagenesis following manufactures’s instruction (Agilent). Primers are: 5’-GAGATATACCATGGGTTGCTCTGGTCTGAACGAC-3’, and 5’- GTCGTTCAGACCAGAGCAACCCATGGTATATCTC-3^’^, useng pET28a-ApqZ as template [48]. The modification was verified by DNA sequencing.

#### 2.1.4. AquaporinZ Expression and Purification

The constructed plasmid was transformed into *Escherichia coli* strain C43 (DE3). Single colony was cultured overnight at 37 °C in 5 mL Luria Broth (LB) medium containing 50 μg/mL kanamycin (Thermo Fisher Scientific, Waltham, MA, USA). The overnight culture was then inoculated into 300 mL fresh LB medium with 50 μg/mL kanamycin and shaking at 250 rpm at 37 °C. The cells were induced with 1 mM Isopropyl β-D-1-thiogalactopyranoside (IPTG) (Sigma Aldrich, St. Louis, MO, USA) when the absorbance at 600 nm reached 0.8. After 4 h incubation, the cells were collected by centrifugation at 8000 × *g* for 10 min.

To purify the protein, cell pellet was re-suspended with 30 mL Phosphate buffered saline (PBS) buffer (Thermo Fisher Scientific, Waltham, MA, USA) (20 mM NaPO_4_, 0.3 M NaCl and pH 7.9) supplied with 0.5 mM protease inhibitor phenylmethylsulfonyl fluoride (PMSF, Sigma Aldrich, St. Louis, MO, USA) and sonicated for 20 min on an ice-water bath. The cell lysate was clarified by centrifugation at 15,317× *g*, 4 °C for 20 min. Then cell debris was dissolved using 2% Triton in PBS buffer and incubated with shaking for 2 h at 4 °C to extract membrane protein. The re-suspension was clarified with centrifugation at 15,317× *g*, 4 °C for 20 min and the supernatant was collected. Ni-NTA agarose beads (Qiagen, Germantown, MD, USA) was mixed with the supernatant for 40 min at 4 °C with shaking. The resin was then loaded into an empty column, drained, and washed with PBS buffer supplemented with 0.03% DDM (n-Dodecyl β-D-maltoside, Sigma Aldrich, St. Louis, MO, USA) and 40 mM imidazole (Sigma Aldrich, St. Louis, MO, USA). Protein was eluted with 500 mM imidazole and 0.03% DDM in PBS buffer. Imidazole was removed by dialysis against PBS buffer supplemented with 0.03% DDM overnight.

An inactive mutant Aqp-SH R189A was also expressed, according to previously published protocol [11], to be used as a negative control to the -Cys modified Aqp. In order to express the inactive mutant, the arginine (R) residue at position 189 in AqpZ was replaced with alanine (A) using site-directed mutagenesis. The Arginine constriction region in aquaporins not only provides the selectivity filter but it also ensures that proton transport is blocked. The replacement of Arginine with Alanine causes a proton transport through aquaporins [49]. This inactive mutant of aquaporin shows no selectivity towards water [50]. Hence, it was used to prepare a negative control of the functional Aqp-SH modified membrane. The protocol used to incorporate Aqp-SH R189A was the same as that used to incorporate functional Aqp-SH into PBI membranes.

### 2.2. Methodology

#### 2.1.1. PBI Membranes Casting

PBI membranes were prepared according to previously published studies [40,43]. The dope solution was diluted to 21% PBI by adding solvent. The non-solvent phase that was used in this process was water. Flat sheet membranes were prepared using casting knife, or doctor’s blade (Paul N Gardner Co, U.S. Pat 4869200, Pompano Beach, FL, USA) The membranes were stored in a 50/50 glycerol-DI water solution in order to prevent their drying and collapse of pore structure. The membranes were kept in the solution at least one day before they were analyzed.

#### 2.2.2. Surface Activation of Membranes

In order to attach Aqp-SH and PVA-alkyl to PBI membranes, membrane surfaces were activated following previous techniques [40,43], in which 4-chloromethyl benzoic acid (CMBA) purchased from Sigma-Aldrich (St. Louis, MO, USA) was used in order for functionalization. CMBA added a carboxylic acid group to the surface of PBI membrane, which could be used as a platform for subsequent functionalization of the membrane [41,42].

#### 2.2.3. Preparation of PVA-Alkyl

PVA-alkyl was prepared in a two-step process, according to literature protocols [40,51–53]. Briefly, a reaction between PVA and sodium monochleroacetate (Sigma Aldrich, St. Lou is, MO, USA.) yielded carboxy-methyl PVA (PVA-COOH), and PVA-alkyl was prepared by reacting PVA-COOH with hexadecanal [40,52].

#### 2.2.4. Chemical Attachment of -Cys Modified Aqp to PBI Backbone

**Figure 3.**
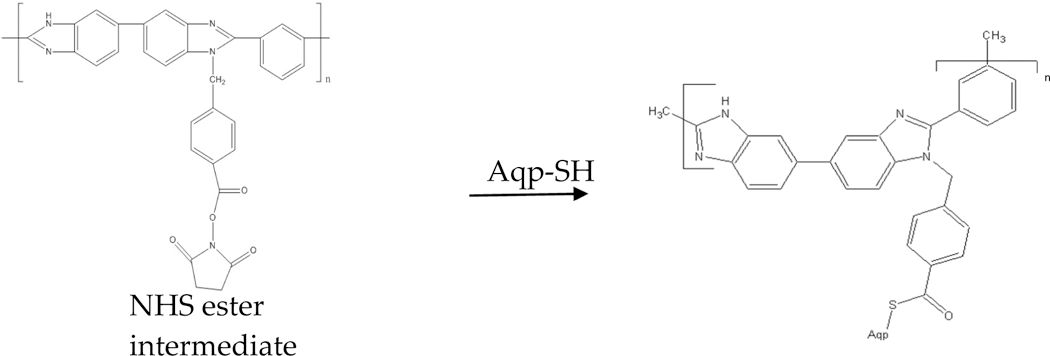
Chemical attachment of Aqp-SH to -COOH modified PBI membranes using; carbodiimide chemistry.

Immobilization of aquaporins into polymer matrix was done in order to align their channels with the direction of water flux and to optimize their performanse. Aquaporins were covalently attached to the modified PBI backbone *w*ith carbodiimide chemistry. For thit task, flat sheet PBI membranes were prepared and modified with CMBA. In the next step, Cys modified Aquaporin (Aqp-SH) were covalently attached in a reducing environment to the -COOH modified PBI membrane using carbodiimide chemistry, as shown in Figure 3. In this mechanism, Aqp-SH acted as a nucleophile to get covalently attached to the -COOH group present on the surface of PBI membrane. Cys groups present after the N-terminus acted as anchors the Aquaporin molacules to prevent the swaying and to help with the alignment of the aquaporin molecules in the direction of the flow. PVA-alkyl was; used in order to bind to the remaining -COOH groups present in the membrane and la seal the gaps in between the attached Aqp-SH molecules following the EDCH chemistry previously used.

##### 2.2.5. Surface Modification of PBI Membrane Using PVA-Alkyl

PVA-alkyl was attached to the membrane using carbodiimide chemistry as reported in previous studies [40],

#### 2.2.6. Membrane Characterization

##### Dynamic Light Scattering

Since aquaporins form the functional element of biomimetic membranes, producing high quality proteins is critical. Before immobilizing proteins on membrcne surface, it is important to evaluate the proteins for their concentration, purity and aggregation state. For this purpose, analysis of protein solution with dynamic light scattering to determine the: presence and extent of aggregation was carried out in Litesizer 500 particle analyzer by Anton Paar (Ashland, VA, USAy An aquaporin solution was taken in a glass cuvette and a plot of particle size vs relative frequency and polydispersity index (PDI) of the solution was obtained. Good quality protein samples would have PDI of 0.08, acceptable quality protein would have PDI of 0.1 to 0.4, while the precipitated protein would have PDI of 0.4 to 0.9 [19].

##### Molecular Weight Cut Off (MWCO)

The MWCO analysis of unmodified PBI, CMBA modified PBI (PBI-CMBA), and PVA-alkyl modified PBI membranes was conducted using 100 ppm solutions of various molecular weights of polyethylene glycol (PEG) and sucrose solutions. The total organic carbon (TOC) of both feed and permeate solutions were measured using Teledyne Tekmar Fusion TOC analyzer (Mason, OH, USA). The various samples that were used in this study along with their Stokes-Einstein radii are shown in Table 1. The rejection values of all solutes were used to determine the MWCO of both unmodified and modified PBI membranes. The molecular weight of solute in feed solution for which the membranes showed more than 90% rejection was considered the MWCO of the membranes. The apparent solute rejection R (%) was calculated using (Equation 1).

(1) $$\text{R}=\left(1-\frac{{\text{C}}_{\text{p}}}{{\text{C}}_{\text{f}}}\right)\times 100\%$$

where C_p_ and C_f_ are solute concentrations in permeate and feed solutions respectively.

##### Contact Angle Measurements

Contact angle was used as a measure to determine the hydrophilicity of the membrane surface. A drop shape analyzer—DSA 100 (KRUSS USA, Matthews, NC, USA) was used for contact angle measurements using sessile drop technique.

##### Zeta Potential and Surface Charge Analysis

Zeta potential is used to analyze the surface charge of membranes at different pH environments. It is particularly important to analyze the separation efficiency of membranes based on charge and also a confirmation test for surface modification [59]. Surface charge was analyzed by measuring the zeta potential using an Anton Paar SurPASS electrokinetic analyzer (Anton Paar, Ashland, VA, USA) in surface analysis mode. Before analysis, membranes were rinsed with copious amounts of DI water to remove any residual solvent or glycerol from the storage solution in the case of PBI membranes. The KCl electrolyte solution (Sigma Aldrich, St. Louis, MO) used in these measurements had an ionic strength of 1.0 mM. The pH values for the various readings were adjusted using 0.5 M HCl (Sigma Aldrich, St. Louis, MO, USA) and 0.5 M NaOH (Sigma Aldrich, St. Louis, MO, USA) solutions for acid and base titrations.

##### Elemental Analysis

Membranes modified with Aqp-SH were analyzed for changes in the concentration of sulfur since unmodified PBI, -COOH modified PBI, and PVA-alkyl modified PBI membranes do not contain any sulfur present in their structures. Hence, Aqp-SH modified membranes were analyzed for the sulfur concentration in them as a confirmation for attachment of aquaporins to the membranes. K-Alpha x-ray photoelectron spectrometer (XPS, Thermo Fisher Scientific, Waltham, MA, USA) was used in order to analyze the elemental composition along the cross section of both unmodified and Aqp-SH modified membranes. Depth profiling was performed using an ion beam to etch layers of membrane surfaces and elemental composition was measured after each etching cycle. An ion beam of 200 eV was used to etch the surface. Three etching cycles were performed for 120 s each for elemental analysis along cross sections of membranes.

##### Membrane Morphology

To investigate the cross-section of the membrane and measure the thickness of selective layer of modified membrane, ion beam of the FEI Helios Nanolab Dual beam was used to cut out a small piece of the membrane. A small deposit of platinum with a thickness of around 60 nm was deposited over the area in order to protect the underlying surface during the process of cutting of cross-section by ion beam. A small cross section was cut out and lifted away from the rest of the membrane sample by welding a small bead of platinum to the platinum layer. This sample was then thinned out with a low power ion beam until the morphology of the mesoporous layer was visible using STEM mode in the Dual Beam. This sample was transferred into the JEOL 2010F (Peabody, MA, USA) for TEM imaging of the cross-section.

##### Flux Analysis

Flux profiles of PBI, PVA-alkyl modified PBI, inactive Aqp-SH modified PBI, and active Aqp-SH modified PBI membranes (called just Aqp-SH modified membranes) were obtained using dead end filtration in an Amicon filtration cell (Amicon Stirred Cell 8010—10 mL, Burlington, MA, USA) under a constant pressure of 0.48 MPa (4.83 bar) and continuous stirring. Flux values were calculated and plotted against the total permeate volume. Membrane samples were cut into circular pieces of area 4.1 cm^2^ and supported by a Whatman™ filter paper (125 mmø, Sigma Aldrich, St. Louis, MO, USA). Each membrane was precompacted with DI water for 1 h until a stable flux was reached.

Precompaction was followed by feed solutions of monovalent and divalent salt solutions in water under same conditions of pressure and stirring. Salt rejection was evaluated using five solutions of different concentrations of sodium chloride (NaCl, Sigma Aldrich, St. Louis, MO, USA) and calcium chloride (CaCl_2_, Sigma Aldrich, St. Louis, MO, USA) in DI water: 3.4,10,20, 35 and 100 mM solutions. Salt concentrations were measured using conductivity meter. Solute rejections were calculated using Equation (1).

After each feed water filtration, reverse flow filtration using DI water was performed for 1 h to remove reversibly-attached foulants that were not adsorbed to the membrane and the filter paper support was changed. The flux recovery of the membrane was measured afterwards in order to study the effect of presence of aquaporins on removal of reversible fouling.

In order to analyze linearity of DI water flux through unmodified and Aqp-SH modified PBI membranes, flux values were measured using DI water as feed solution at four different pressure values: 1.38, 2.76, 4.14, and 5.52 bar.

Unmodified and modified membranes were subjected to dead end flow filtration using 0.5 M, 1 M, and 2 M NaCl and CaCl_2_ solutions in order to compare the rejection trends of membranes under high salt concentration feed solutions. Inductively coupled plasma (ICP) analysis was used to measure the concentrations of permeates obtained from all membranes.

#### 2.2.7. Estimation of Aquaporin Packing in Membrane Assembly:

Membrane porosity and double layer properties influence ion fluxes through the membrane. The flux values measured for Aqp-SH modified membranes exhibited weak sensitivity to ionic strength. These fluxes (j) can be estimated via an ion’s concentration (c) gradient and its diffusion coefficient (D), as shown in Equation (2):

(2) $$\;\text{j=}\;\text{D}{▽}_{\text{C}}$$

assuming a concentration gradient was imposed perpendicular to a porous film. This concentration gradient was set by the ion concentrations in reservoirs to either side of the membrane as well as their separation. By relating the measured flux to the concentration gradient, an effective diffusion coefficient, De, could be determined. This allowed the inference of relative packing densities of aqp molecules incorporated in the active layer of membrane. This effective diffusion coefficient would be generally smaller than the ion’s intrinsic diffusion rate in bulk media, and moreover, it would be proportional to the ratio of the accessible pores’ surface area to the total surface area, assuming the channels were perfectly linear and aligned with the concentration gradient, e.g., ${\text{D}}_{\text{e}}=\frac{{\text{SA}}_{\text{pore}}}{{\text{SA}}_{\text{total}}}\times \text{D}$. According to the SEM imaging data of cross-sections of membranes published previously [40], it was further assumed that the PVA-alkyl and PBI were stacked in layers aligned perpendicular to the concentration gradient.

Based on these assumptions, a numerical partial differential equation was used to estimate how ionic fluxes were modulated by aquaporin surface densities, from which aquaporin packing densities compatible with experimentally-measured flux data could be determined. Namely, finite element simulations of the steady state Fickian diffusion Equation (3) were performed,

(3) $$\frac{\text{dc}}{\text{dt}}=-\nabla \text{j}$$

subject to c(L) = 1 mM and c(R) = 0 mM, where c is the concentration of the ionic solution, D is the diffusion coefficient and L, R correspond to the left and right reservoir boundaries. From these simulations, an effective diffusion coefficient that reflected the impact of the channel geometry on transport was determined. This proceeded through recognizing the flux was related to the concentration gradient via Equation (4)

(4) $$<j>=\frac{1}{A}\int \text{D}\;{▽}_{\text{c}}\text{dS}$$

where A is the surface area of the film and S represents the surface. Flux could be expressed in terms of concentrations and De was given by Equation (5)

(5) $$<\text{j}>\text{A}\sim {\text{D}}_{\text{e}}\frac{\left(\text{c}(\text{L})-\text{c}(\text{R})\right)}{\left(\text{x}(\text{L})-\text{x}(\text{R})\right)}$$

where c(i) is the concentration at boundary i (left and right) and x(i) is the position of the boundary. By numerically evaluating <j> at the film boundary, the equation was solved for De based on the concentrations imposed at the reservoir boundaries and their separation distance.

**Figure 4.**
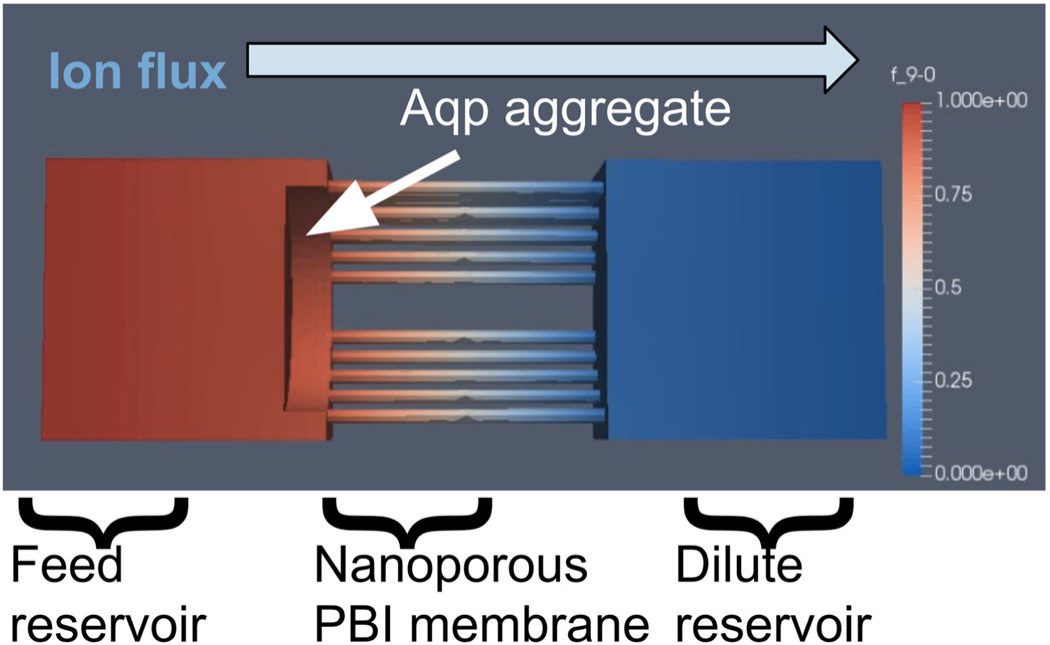
Representative simulation geometry of the membrane occluded by aquaporin (Aqp) aggregates. Here the left reservoir contained 1000 ppm NaCl solution versus pure solvent in the right reservoir (0 M NaCl). Aqp aggregate-size was simulated by cylinders of increasing diemeter overlaid onto the membrane surface. Packing; density ‘was also tuned by changeng the relative area of the PBI membrane. The effective ion diffusion rate was obtained by integrating the; concentration gradient along the membrano surface, based on numerical simulation of the steady-state diffusion equation on this geometry.

These equations were solved on three-dimensional finite element meshes [59,60], based on potential membrane and aquaporin configurations using the mesh generation tool GMSH [59,61] (See Figure 4). The meshes consisted of two reservoirs separated by a porous domain representing the film. Aqp or aggregates thereof were represented by cylinders of varying radii aligned parallel to the membrane. In principle, atomistic resolution surface geometries could have been used for the Aqp molecules [59,60], but since specific knowledge of the membrane structure at the solvent/membrane interface was not known, a simple cylindrical representation of the protein was used. These equations were solved, assuming Dirichlet conditions of c = 1.0 M and c = 0.0 M on the left and right reservoir boundaries [59,60] via the finite element method using FEniCS [59,62]. Thebulk diffusion coefficient was arbitrarily set to D = 1.0 m^2^/s, since we present effective diffusion constants that are normalized with respect to the bulk value. Specifically, the weak form of these equations was solved using a piecewise linear Galerkin basis with FEniC’s default direct linear solver and parameters. Concentration fluxes were determined by performing an ‘assemble’ call on an immersed boundary located at the middle and oriented parallel of the porous film. Details of the numerical procedure follow from previously published work [59,60]. To capture the behavior of monomeric AqpZ, the flux found at the boundary of a pore was normalized [59,60]. The packing fraction observed in the boundary layer then represented a boundary condition surrounding individual aquaporins. All code written in support of this study is publicly available [60]. Simulation input files and generated data are available upon request.

## 3. Results

### 3.1. Dynamic Light Scattering

**Figure 5.**
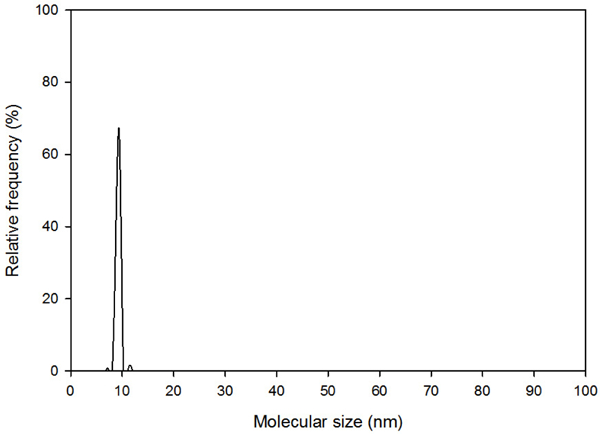
Size measurement of Aqp-SH by dynamic light scattering (DLS). DLS studies showed that aquaporins were not aggregated in the solution.

As shown in Figure 5, the particle size analysis of Aqp-SH solution obtained showed a sharp peak around 10nm, which is the size range of aquaporin molecules and the bound detergent. In addition, the PDI obtained from the particle analyzer was 0.19. This showed that the proteins were not aggregated in the solution and the PDI of aquaporin solution was in the acceptable range [9].

### 3.2. MWCO Analysis

**Figure 6.**
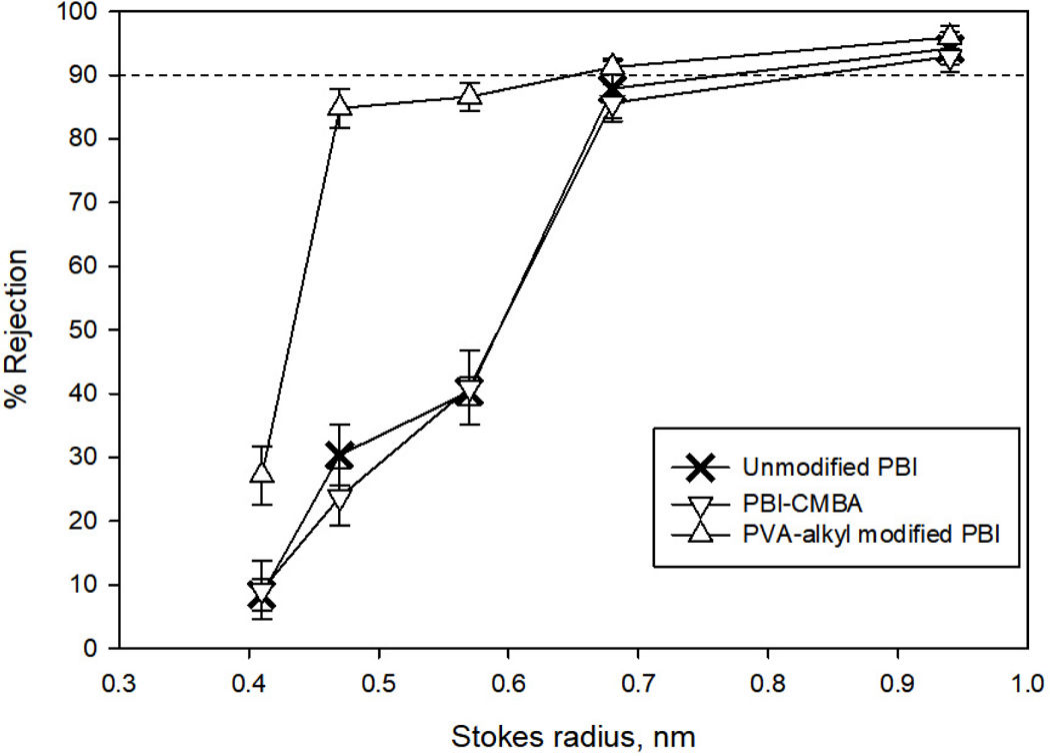
MWCO analysis of unmodified PBI, -COOH modified PBI, and PVA-alkyl modified PBI membranes.

Both unmodified PBI and PBI-CMBA membranes showed 90% rejection for PEC 1000 (Figure 6 and Table 2), which has a Stokes radius of 0.94 nm. This showed that the membranes were in the nanofiltration range. After modification with PVA-alkyl attachment to the membrane, the produced membranes showed a 90%o rejection for PEG 600, which has a Stokes radius of 0.68 nm. This showed that PVA-alkyl modified membranes were also nanofiltration membranes but with smaller pores. Aqp-SH modified membranes showed rejections of 95.2% ± 3.7%, 97.2% ± 1.4% 98.4% ± 0.4% for PEG 200, Sucrose, and PEG 400. In addition, these modified membranes showed complete rejections for PEG 600 and PEG 1000.

### 3.3. Aquaporin Attachment Verification through Elemental Analysis

Depth profiling in XPS analysis was performed for both PBI-COOH and Aqp-SH modified PBI membranes in order to prove the change in sulfur concentration in the membranes after modification. Tables 3 and 4 show weight percentage of atoms of carbon, oxygen, nitrogen and sulfur present in the membrane samples. It can be seen from Table 3 that the amount of sulfur is negligible in PBI-COOH membrane, which was expected since the structure of -COOH modified PBI [40,43] does not contain any sulfur. A small amount of sulfur shown in unmodified membrane might be due to impurities present on the surface and polymer matrix of the membrane. Table 4 shows some amount of sulfur in Aqp-SH modified membrane. Each aquaporin monomer contained four cysteine groups including the one attached at the end groups. Considering the tetrameric form of aquaporins, there are 16 sulfur atoms present in one aquaporin molecule. Hence, for a point scan, the amount of sulfur present at a level in Aqp-SH modified membranes should be between 0.5% and 1%. Therefore, elemental analysis of both unmodified and modified PBI membranes showed the presence of sulfur in the Aqp-SH modified membrane.

### 3.4. Hydrophobicity

Contact angle was used as a measure of hydrophobicity, and results are shown in Table 5. CMBA modified membranes were found to be more hydrophilic than PBI membranes [42,43]. This was most likely due to addition of a -COOH group in the modified molecule and its increased ability to form hydrogen bonds because of the presence of oxygen with a lone pair. After the addition of PVA-alkyl to the membranes, the contact angle decreased further showing a significant increase in the hydrophilicity of the membrane. This was most likely due to high hydrophilicity of PVA. It is hypothesized that hydrophobic part of PVA-alkyl was reoriented so that the alkyl chains were inside the membrane matrix whereas PVA was on the outside, thus making the membranes more hydrophilic [61]. After chemical attachment of Aqp-SH and PVA-alkyl, there was no significant difference in the contact angle showing that most of the surface of Aqp-SH membrane might be covered with PVA-alkyl, providing a protection to Aqp-SH. The middle portion of AqpZ is hydrophobic, but the ends are hydrophilic as these parts are exposed to the cytosol/periplasm. In case of aquaporins aligned to the feed direction and exposed to the surface, the hydrophilic ends would be facing up, and this would be responsible for an increase in contact angle if they were exposed on the surface of the membrane [4].

### 3.5. Zeta Potential and Surface Charge Analysis:

**Figure 7.**
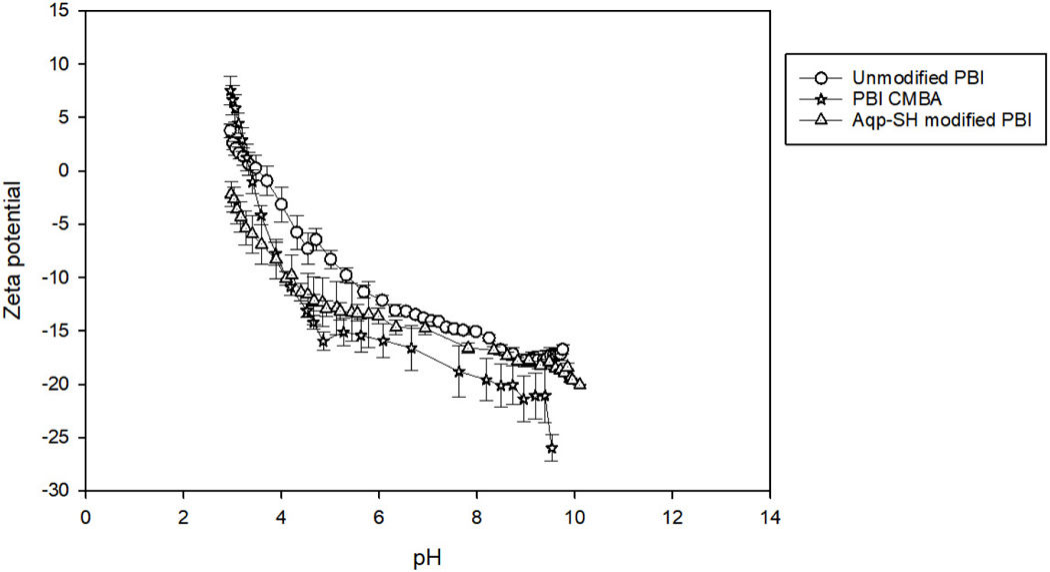
Zeta potential values of unmodified PBI, PBI-CMBA, and Aqp-SH modified PBI membranes over a pH range of 2–10.

Aquaporins have histidine groups present at the pore opening which are positively charged [62]. In order to confirm that the aquaporins were not exposed on the surface of Aqp-SH modified membranes, zeta potential analysis was carried out of unmodified PBI, PBI-CMBA, and Aqp-SH modified PBI membranes over a pH range of 2–10 (Figure 7). There were no significant differences between the surface charge curves of the three membranes, with PBI-CMBA showing a more negative trend as compared to the others likely due to the additional of functional carboxylic end groups to the membrane surface. Since Aqp-SH membranes did not show more positive trends, and in fact showed no significant difference in zeta potential as compared to the unmodified PBI membranes, it is concluded that aquaporins were not exposed on the surface of the membranes.

### 3.6. Membrane Morphology

**Figure 8.**
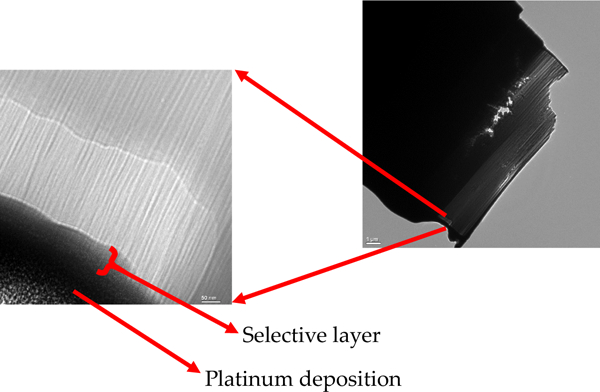
Cross sectional image of a cut out of the modified membrane obtained using TEM. The cutout was obtained using; a focused ion beam instrument.

Membranes modified with Aqp-SH-PVA-alkyl showed a selective layer of approximately 50 nm. However, the cross-sectional images (Figure 8) did not provide any visual confirmation of Aquaporins present in the selective layer of the membrane. A selective layer as thick as 50 nm might be because of the surface modification of PBI membrane with PVA-alkyl. Lack of any visual confirmation of aquaporins in the selective layer might be because there were no vesicles in the system and aquaporins were present in the modification layer as individual molecules surrounded by hydrophobic and hydrophilic groups of PVA-alkyl.

### 3.7. Flux Analysis

**Figure 9.**
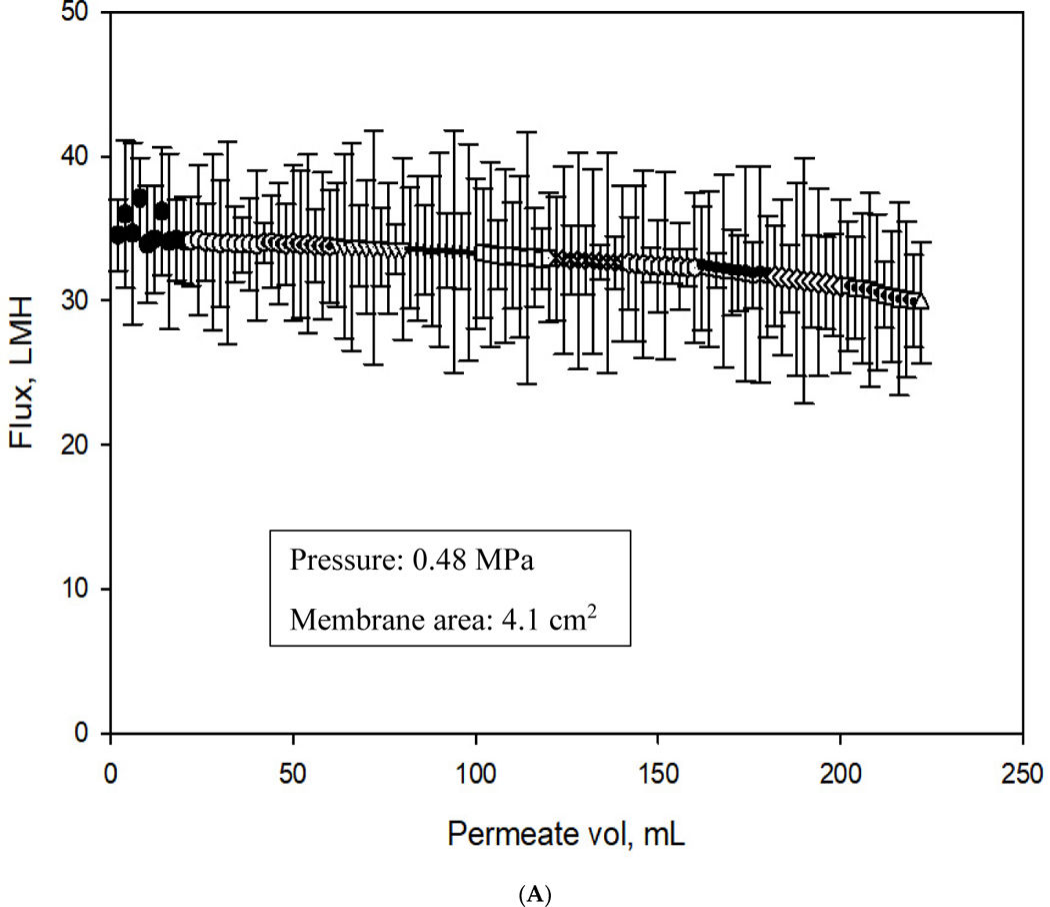

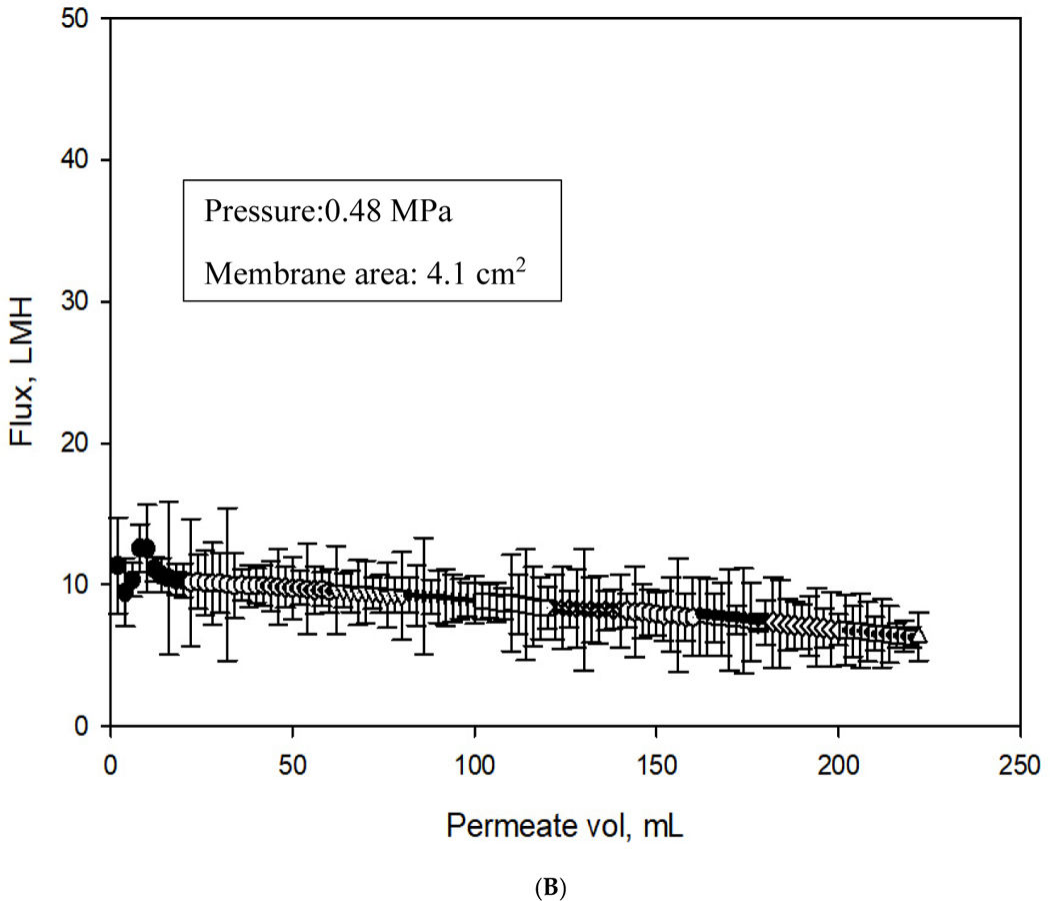

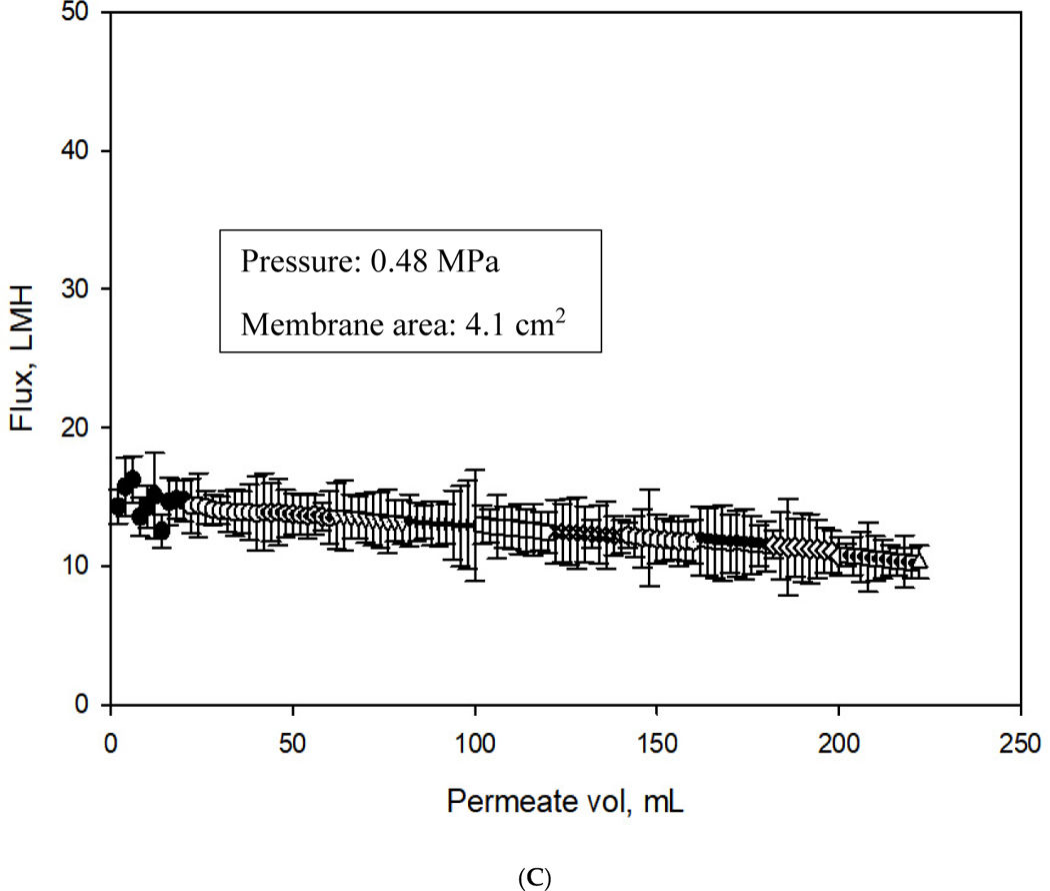

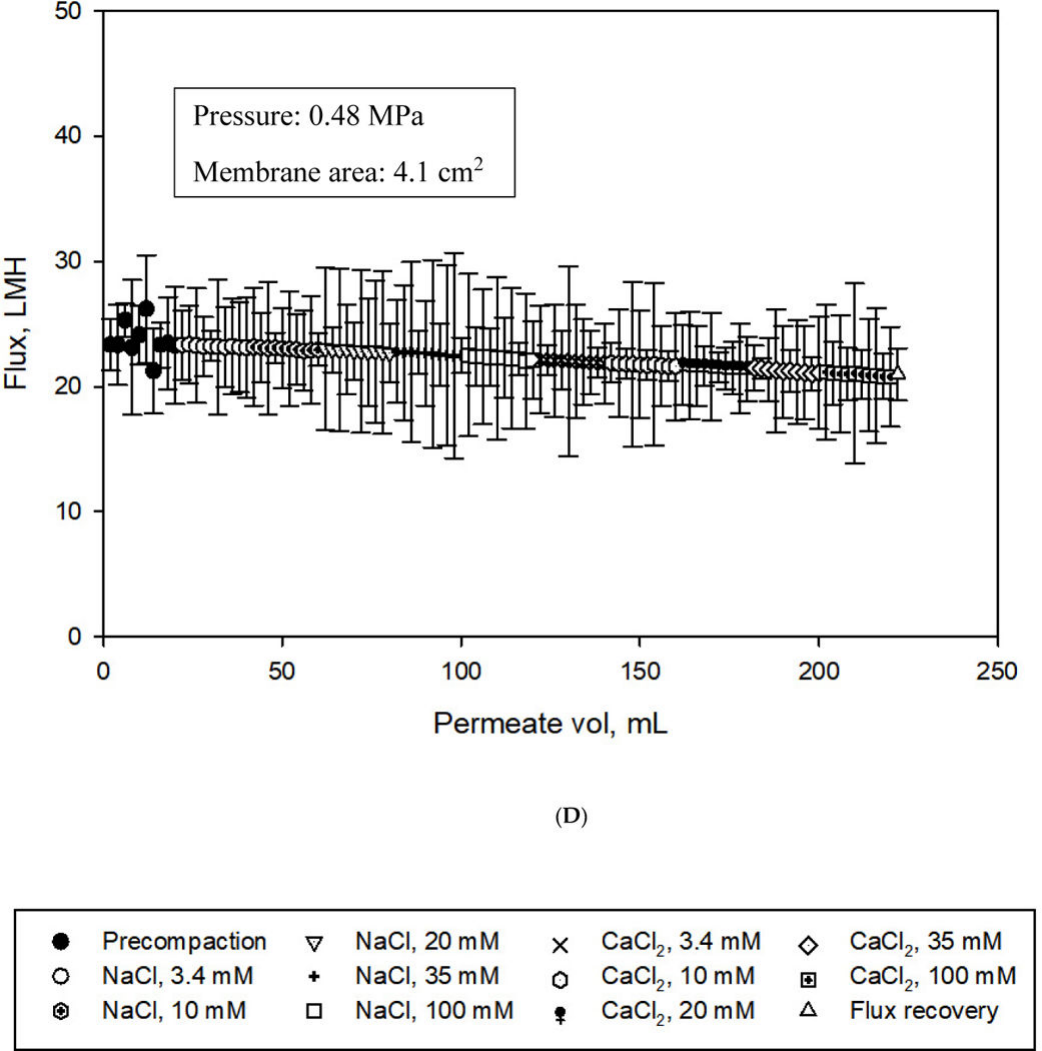
(**A**): Flux analysis of unmodified PBI membrane; (**B**): Flux analysis of PVA-alkyl modified PBI membrane; (**C**): Flux analysis of inactive Aqp-SFl modified PBI membfane; and (**D**): Flux analysis of Aqp-SH modified PBI. All flux analyses were carried out: at constant pressure of 0.48 MPa (4.83 bar). Five concentrations each of NaCl and CaCl_2_ solutions were used as feed solutions and reverse flow filtration was used after every solution filtration.

To investigate the ability of the Aqp-SH membranes to reject ions, filtration studies using different concentration solutions of NaCl and CaCl_2_ in water were performed under a constant pressure of 4.83 bar. Experiments were conducted in parallel in order to study unmodified and modified membranes, and tine flux values were plotted as a function of permeate volume for unmodified, PVA-alkyl modified PBI (to reflect: the amphiphilic matrix without aquaporins), inactive Aqp-SH modified PBI (i.e., to be used as a negative control) and Aqp-SH membranes, as shown in Figure 9A–D, respectively.

PVA-alkyl modified membranes showed the lowest initial flux, filtration flux and recovered flux among all membranes possibly because oh added resistance to flow due to tine addition of *a* dense layer to the surface and because; of a decrease In pore size (Figure 6). Unmodified PBI membranes showed highest initial flux values, which might have been due to the absence of any layer adding resistance on the surface of membranes. The flux profile obtained for the inactive Aqp-SH membranes did not show any significant change when compared to that of PVA-alkyl modified membranet, possibly due to the lafk of water permeability of inactive mutant ol aquaporins (Aqp-SH R189A) [11,50]. The incorporation of aquaporins on PVA-alkyl modified membranes showed an increase in flux values as compared to PVA-alkyl membranet as well as the membranes modified with inactive mutant; however, the flux values of Aqp-SH membranes were still lower than those of unmodified PBI membranes. The addition of PVA-alkyl alone acted to both block pores and increased resistance to flow, and hence, decreased flux. The addition of functional aquaporins to these membranes provided them with flow channels, which increased the flux as compared to PVA-alkyl membranes. However, the flux was not as high as the modified membranes owing likely to the fact that aquaporin coverage was not complete over the surface of the PVA-alkyl, so there were still regions of minimal or no flow. Additional experiments were conducted in order to analyze the flux linearity of unmodified and modified membranes. Fluxes produced by all the membranes increased linearly with increment in pressure. Also, the incorporation of immobilized aquaporins and dense PVA-alkyl layer on the surface of PBI membrane did not affect the flux linearity of the membranes (Figure S1).

**Figure 10.**
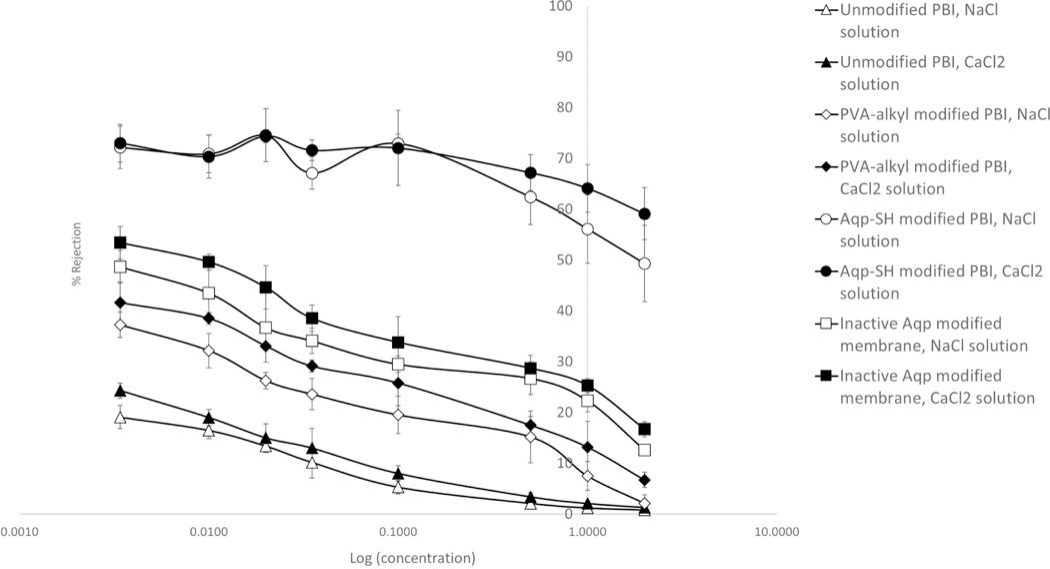
Rejection trends for Sodium chloride and calcium chloride filtration.

With respect to salt rejection (Figure 10), Aqp-SH membranes showed the highest rejections for the solutions as compared to unmodified PBI and PVA-alkyl modified PBI membranes. Unmodified PBI membranes showed 19 ± 2.3% rejection during filtration of the 3.4 mM NaCl solution, and as the NaCl concentration increased to 100 mM, the rejection decreased to 5.3 ± 1.2%. PBI membranes modified with only PVA-alkyl showed a rejection of 37.24 ± 2.5% for a feed solution of 3.4 mM NaCl solution and 19.53 ± 3.7% rejection for 100 mM NaCl solution. PBI membranes modified with inactive mutant of Aqp (Aqp-SH R189A) showed 48.7 ± 3.2% rejection during filtration of the 3.4 mM NaCl solution, and as the NaCl concentration increased to 100 mM, the rejection decreased to 29.5 ± 5.1%. On the other hand, Aqp-SH membranes showed a significantly higher rejection of 72.15 ± 4.2% for 3.4 mM feed solution of NaCl and 72.95 ± 1.8% for 100 mM NaCl. Similarly, unmodified PBI membranes showed 24.30 ± 1.5% rejection during filtration of the 3.4 mM CaCl_2_ solution, and as the CaCl_2_ concentration increased to 100 mM, the rejection decreased to 8 ± 1.8%. PVA-alkyl modified PBI membranes showed 41.61 ± 4% rejection for 3.4 mM CaCl2 and 25.82 ± 4.5% rejection for 100 mM CaCl2. Aqp-SH R189A modified PBI membranes showed 53.4 ± 3.2% rejection for 3.4 mM CaCl2 and 33.8 ± 1.6% rejection for 100 mM CaCl_2_. On the other hand, Aqp-SH membranes showed a rejection of 73.01 ± 3.7% for 3.4 mM feed solution of CaCl_2_ and 72.0.4 ± 7.4% for 100 mM CaCl_2_. To demonstrate the effectiveness of the functionalizing aquaporins with cysteine end groups (i.e., Aqp-SH), results were compared to those using the exact same membranes (PVA-alkyl modified PBI membranes) with regular aquaporin Z (AqpZ) added to them [40]. As non-functionalized AqpZ does not have cysteine groups as anchors to attach chemically on membrane surface, the non-functionalized AqpZ were added via physical incorporation into PVA-alkyl, which acted as a surface modification layer on PBI membrane. Results showed that membranes modified with aquaporins displayed lower flux declines and higher flux recoveries after reverse flow filtration, along with improved rejection values for both protein and salt solutions as compared to PBI and PBI-PVA-alkyl membranes. However, there was leakage of ions between channels as observed by salt rejections decreasing as a function of feed concentration, from approximately 70% at 10 mM to less than 40% at 100 mM. On the other hand, membranes modified with functionalized aquaporins (Aqp-SH) showed consistently higher salt rejection values of ~70% irrespective of feed concentration, along with higher flux recoveries and lower flux declines.

All membranes were then subjected to high concentration feed solutions of NaCl and CaCl_2_ (Figure 10). Unmodified PBI membranes showed 2.1 ± 0.5% rejection during filtration of the 0.5 M NaCl solution, and as the NaCl concentration increased to 2 M, the rejection decreased to 0.8 ± 0.4%. PBI membranes modified with only PVA-alkyl showed a rejection of 15.21 ± 5.1% for a feed solution of 0.5 M NaCl solution and 2.13 ± 1.7% rejection for 2 M NaCl solution. Aqp-SH R189A modified PBI membranes showed 26.7 ± 2.6% rejection during filtration of the 0.5 M NaCl solution, and as the NaCl concentration increased to 2 M, the rejection decreased to 12.6 ± 1.5%. On the other hand, Aqp-SH membranes showed a significantly higher rejection of 62.4 ± 5.4% for 0.5 M feed solution of NaCl and 49.3 ± 7.5% for 2 M NaCl. Similarly, unmodified PBI membranes showed 3.4 ± 0.8% rejection during filtration of the 0.5 M CaCl_2_ solution, and as the CaCl_2_ concentration increased to 2 M, the rejection decreased to 1.3 ± 0.2%. PVA-alkyl modified PBI membranes showed 17.52 ± 1.7% rejection for 0.5 M CaCl_2_ and 13.19 ± 5.1% rejection for 2 M CaCl_2_. Aqp-SH R189A modified PBI membranes showed 28.7 ± 3.2% rejection during filtration of the 0.5 M CaCl_2_ solution, and as the CaCl_2_ concentration increased to 2 M, the rejection decreased to 16.7 ± 1.0% On the other hand, Aqp-SH membranes showed a rejection of 67.2 ± 3.5% for 0.5 M feed solution of CaCl_2_ and 59.1 ± 5.1% for 2 M CaCl_2_.

PVA-alkyl modified membranes showed higher salt rejection values as compared to unmodified PBI membranes possibly due to a decrease in MW CO, pore size of membranes, and charge interactions with ions. Membranes modified with inactive mutant of Aqp showed slightly higher rejections than PVA-alkyl modified PBI membranes. However, the difference between PVA-alkyl modified PBI membranes and Aqp-SH R189A modified PBI membranes was not significant. Two different samples were used in order to carry out flux analysis and salt rejection studies. The slight difference between salt rejections obtained from PVA-alkyl modified PBI and Aqp-SH R189A modified PBI might be due to the variation of concentration of PVA-alkyl on the surface of membrane sample. The rejection obtained for Aqp-SH modified PBI membranes for salt solutions were higher as compared to the unmodified PBI membranes. This might be due to the immobilized aquaporins on the membrane surface.

**Figure 11.**
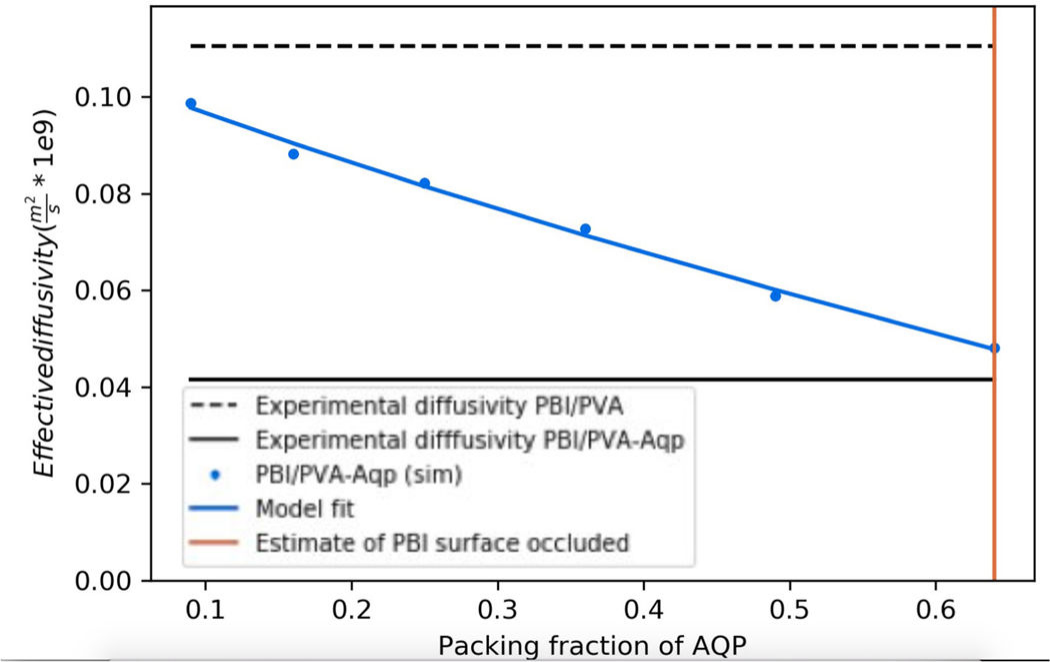
Predictfons and experimentally measured effective diffusion coefficients, based on the geometry in Figure 4, Black lines correspond to experimental dada found in Supplemental Table S1. Blue lines represent aggregate (solid) and monomeric (dots) Aqp models, respectively.

Rejection properties are in part determined by the electric double layer that arises from the electrostatic potential about charge d surfaces in aqueous media; the magnitude of this potential and its rate of decay from the surface are determined by the surface charge and ionic strength, respectively [63]. As the ionic strength of feed solution increases, rejection decreases owing to *a* contraction of the electric double layer that enhances charge shielding [64,65] and reduces ion transport rates [66]. However, in tire case of aquaporin modified membranes, membrane rejection remained fairly constant irrespective of the ionic strength oi salt solutions (Figure 11). In the case of unmodified PBI membranes and membranes modified with PVA-alkyl, a decrease in salt rejection was observed as the ionic strength of feed salt solutions increased, which corresponds lo double layer and charge shielding effects. The constant rejecter- of salt solution obtained with aquaporin modified membranes shows that immobilized aquaporins might be unaffected by charge interactions and provide the same rejection, irrespective of the ionic strengths of the feed solutions. The reason might be because of the unique hourglass shaped structure of aquaporin pores and the electrostatic barrier able lo reject all the charged entities present in feed other than water molecules [67,68], which means that any decreases in rejection would be due to leakage around the aquaporins. The free-energy profile for ion penetration through aquaporin modified membrane shows a significant difference between the overall barriers for ion add water penetration. The constant rejection observed with Aqp-SH modified membranes is another evidence of the presence oi functionalized aquaporins that opened up more water channels, increased water flux through the membrane, end provided higher and constant rejection of feed salt solutions irrespective of their ionic strength. However, it is likely that aquaporins did not cover entire surface area of the membranes due to the presence of detergent or PVA-alkyl, so some of the feed solution might have gone around the aquaporins on the surface, providing a rejection less than the complete rejection that was expected with aquaporins.

### 3.8. Estimations of Aquaporin Packing in Membrane Assembly

Although the polymer layers could be resolved via electron microscopy, the distribution of surface-anchored Aqps were beyond limits of detection. Thus, to investigate the hypothesis that aquaporin surface deposition was incomplete, a computational model was used to measure ion fluxes across a membrane with aquaporins aggregates of varying sizes. In principle, complete coverage of the film surface with aquaporins should reduce electrolyte flux across the membrane to zero while permitting water flux owing to the high selectivity of aquaporins to water over ions. Although electrostatic interactions with charged surfaces can strongly influence ion conduction [69], the high ion concentrations at which the experiments were conducted strongly attenuated such effects.

To rationalize the flux values reported in Figure 9A–D that demonstrated significant variations in magnitude with respect to polymer membrane configuration, a computational geometry was developed. The 1 nm diameter pores were consistent with MWCO analysis; the pore spacing accounted for 28% surface coverage by nanopores. For this modeling, the PVA-alkyl porosity was assumed to be invariant across the characterized membrane configurations. Since ions do not permeate through the Aqp pore, the proteins were presumed to comprise a monolayer of cylindrical obstructions that resist flow through the PBI layer. Here it was assumed that Aqps capped the PBI-pores and the 64% reduction in flux reported in Figure 9B for PVA-alkyl-only relative to Aqp-SH modified membranes could be attributed to capping a commensurate percentage of available pores. However, these data are insufficient to density of the channels on the membrane surface.

Using a computational simulation of electrolyte diffusion through the Aqp-studded membranes (Figure S2; Table S1), we investigated the extent to which electrolyte fluxes at the membrane were influenced by the Aqps distribution: either as single proteins distributed uniformly or as aggregates. Hence, to determine whether the Aqp-SH behave as aggregates or uniformly distributed channels, we resorted to a three-dimensional, partial differential equation-based model that could account for a range of possible Aqp-SH distributions and packing densities. The model largely follows from our implementation described in previous studies [66,70] and we include implementation details in Methods Section 2.2.7.

Toward this end, the steady state diffusion equation was solved based on varying Aqp aggregate sizes. Figure 11 shows a disk of increasing radius that occluded the underlying pores as a representation of Aqp-aggregates of increasing size. In Figure 10, we demonstrate that the model predicted an increase in effective diffusion rate as the Aqp packing density approached 0, such that the faster diffusion observed experimentally for the PBI-only case was recovered. In other words, NaCl diffusion was not impeded by Aqp and diffused at rates typical of a PBI-only membrane. As the Aqp packing density approached 64%, the effective diffusion constant approached the experimental estimate for the Aqp-modified membranes, which we indicate in Figure 10 by the red vertical line. We additionally show in Figure 10 (model fit) the change in De with respect to packing density for a single Aqp monomer, by varying the surface area of the film for a fixed Aqp monomer size 0.4 nm x 0.4 nm. We simulated the effect of varying the monomeric Aqp packing fraction, by scaling the average concentration flux at the Aqp over a range of surface areas, as this flux will scale proportional to the occluded surface area. It was found that the effective diffusion rate scaled comparably to the aggregates, hence these two cases could not be discriminated based on diffusion alone.

It is important to note that Aqp monomers at a given packing density presented more exposed surface area compared to an aggregate of comparable density. In light of which, packing configurations could be discriminated under conditions that manifest strong surface/diffuser interactions. For instance, in the event that these experiments were performed under low ionic strength conditions, it was expected that ions could interact with the Aqp surface and thereby influence diffusion, either through weak electrostatic interactions or high affinity binding [69]. In addition, as shown in in Figure 9D, the initial flux obtained for Aqp-SH modified membranes could be due to the aggregation of some of the aquaporins on membrane surface. Using; the experimental flux data obtained in Figure 9D, the packing fraction of dispersed aquaporins was found to be ~64 %.

## 4. Concluding Remarks

Modification of aquaporins with a cysteine at the N-terminus and immobilization of these modified aquaporins on the membrane surface was successfully accomplished. Elemental analysis showed that aquaporins were immobilized on the membrane surface. It is proposed that four cysteine groups acting as anchors for aquaporin tetramer helped to align aquaporins on the surface of membranes. In agreement with pore size distributions, charge interactions, and added resistance to flow due to modification, PVA-alkyl modified PBI membranes showed lower flux values and slightly higher salt rejection as compared to unmodified PBI membranes. On the other hand, Aqp-SH modified membranes displayed lower flux values; compared to unmodified PBI but higher as compared to PVA-alkyl modified membranes. Membranes modified with an inactive Aqp-SH were used as a negative control to demonstrate the functionality of Aqp-SH incorporated into tire membranes. Inactive Aqp-SH modified membranes did not show any improvement in flux values as compared to PVA-alkyl modified PBI membranes. Furthermore, owing; to the presence of functional and immobilized aquaporins, Aqp-SH modified membranes displayed the highest salt rejection values among all membranes analyzed in the study. Aqp-SH modified membranes displayed a nearly constant salt rejection irrespective of the salt concentration for low feed concentration, while unmodified PBI and PVA-alkyl modified PBI membranes showed a decrease in rejctions as feed salt concentration increased. However, due to the hindrance of detergent or PVA-alkyl in aquaporin solutions, the surface of the membrane was not completely covered with immobilized add aligned aquaporins, which in turn led to rejection values lower than 100%o. Simulation studies showed. that immobilize aquaporins with PVA-alkyl provided a diffusion rate equivalent to 64%_f_ coverage. This proved that aquaporins did not cover the entire surface area of the membranes, thereby providing a salt rejection of less than 100%_t_. In addition, the flux obtained with Aqp-SH modified membranes was lower as compared to unmodified PBI membranes. This might be due to aggregation of some of the aquaporins added onto membrane surface. The packing fraction for dispersed aquaporins on membrane surface was calculated to be ~24%.

## Supplementary Material

supp

## Figures and Tables

**Table 1.** Neutral solutes used for Molecular Weight Cut Off (MWCO) analysis and their Stokes-Einstein radii in nm [54–5,8]. PEG: polyethylene glycol.

Solute	Mol. Wt. (gm/mol)	Stokes-Einstein Radii (nm)
PEG 200	200	0.41
Sucrose	342.3	0.47
PEG 400	400	0.57
PEG 600	600	0.68
PEG 1000	1000	0.94

**Table 2.** Rejections obtained for unmodified Polybenzimidazole (PBI), 4-chloromethyl benzoic acid (CMBA) modified PBI (PBI-CMBA) and aquaporin Z modified with cysteine (Aqp-SH) modified PBI membranes.

Membrane	Rejection > 90%
Unmodified PBI	0.94 nm (94.2% ± 2.5 %)
PBI-CMBA	0.94 nm (93.0% ± 2.4 %)
PVA-alkyl modified PBI	0.68 nm (91.3% ± 1 %)

**Table 3.** Elemental composition of elements in PBI-COOH membrane.

	Carbon	Nitrogen	Oxygen	Sulfur
Surface	85.22	10.7	4	0.07
Level 1	86.28	10.28	3.39	0.05
Level 2	86.2	10.39	3.33	0.09
Level 3	87.3	10.56	2.11	0.03

**Table 4.** Elemental composition of elements in Aqp-SH modified PBI membrane.

	Carbon	Nitrogen	Oxygen	Sulfur
Surface	92.13	4.07	3.3	0.5
Level 1	87.18	8.93	3.41	0.48
Level 2	87.58	8.73	2.99	0.7
Level 3	86.82	8.02	3.05	0.62

**Table 5.** Hydrophobicity via contact angle.

Membrane	Contact Angle
Unmodified PBI	75° ± 0.55
-COOH modified PBI	70.56° ± 1.04
PVA-alkyl modified PBI	60.5° ± 1.44
Aqp-SH modified PBI	57.5° ± 0.93
