# Supplementary material for: Increasing Salt Rejection of Polybenzimidazole Nanofiltration Membranes via the Addition of Immobilized and Aligned Aquaporins
Source: Processes (Basel). Author manuscript; Available in PMC 2019 Jun 5. (PMC6550480; doi:10.3390/pr7020076)
Supplement: supp [file NIHMS1014645-supplement-supp.pdf]

Supplementary Materials

Increasing Salt Rejection of Polybenzimidazole Nanofiltration Membranes via the Addition of Immobilized and Aligned Aquaporins

Priyesh Wagh [1], Xinyi Zhang [2], Ryan Blood [1], Peter M Kekenes-Huskey [1,2], Prasangi Rajapaksha [2], Yinan Wei [2] and Isabel C. Escobar [1,*]

[1] Chemical and Materials Engineering Department, University of Kentucky, Lexington, KY 40506, USA; priyesh.wagh@uky.edu (P.W.); stuart.blood@uky.edu (R.B.); pkekeneshuskey@uky.edu (P.M.K.-H.)
[2] Department of Chemistry, University of Kentucky, Lexington, KY 40506, USA; xinyi.zhang@uky.edu (X.Z.); prasangi_iro@uky.edu (P.R.); Yinan.Wei@uky.edu (Y.W.)
* Correspondence: isabel.escobar@uky.edu; Tel.: +859-257-7990

1. Flux linearity trends:

Additional experiments were conducted in order to analyze the flux linearity of Aqp-SH modified membranes. As shown in Figure 1, the slope of line represents permeability of unmodified, PVA-alkyl modified, and Aqp-SH modified PBI membranes. Unmodified PBI membranes showed a permeability of 6.27 LMH/bar. PVA-alkyl modified membranes showed the lowest permeability of all the membranes at 1.88 LMH/bar, while Aqp-SH modified PBI membranes showed an increased permeability as compared to PVA-alkyl modified membranes (4.46 LMH/bar). This increased permeability might have been due to the presence of immobilized aquaporins on modified membranes providing the modified membrane with more flow channels. Figure 8 shows that fluxes produced by all the membranes increased linearly with increment in pressure. In addition, the incorporation of immobilized aquaporins and dense PVA-alkyl layer on the surface of PBI membrane did not affect the flux linearity of the membranes.

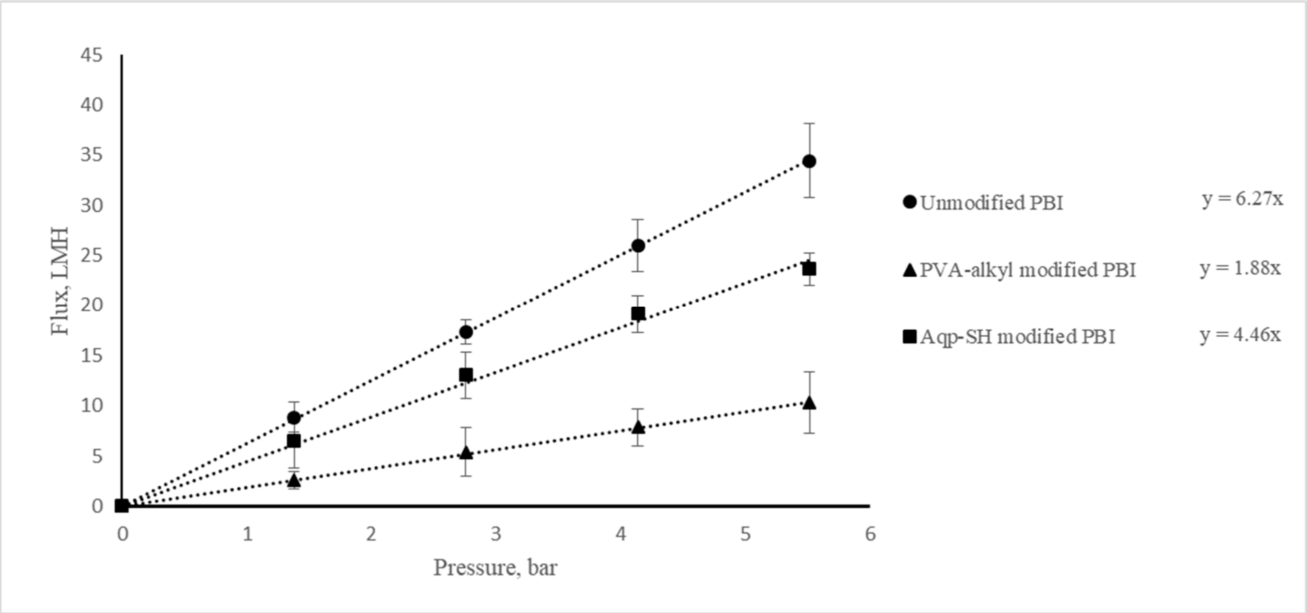

Figure S1. Flux linearity and permeability consistency for unmodified PBI and Aqp-SH modified PBI membranes.

2. Estimation of Aqp packing density:

The properties of nanofiltration lie between non-porous RO membranes where the transport is mainly by solution-diffusion mechanism and ultrafiltration membranes where size exclusion is the dominating mechanism for separation [1]. The extended Nernst-Planck equation forms the basis for the description of the transport of ions/solutes inside the membranes. The equation as written by Bowen et al. [2] is as follows:

$$j_i = -D_i.p \frac{C_i}{dx} - \frac{Z_i C_i D_{i},p}{RT} F \frac{d\psi}{dx} + K_i, cC_i V \quad (1)$$

where, $D_{i,p} = K_{i,d} D_{i,x}$, j_i is the flux of ion *i* and the terms on the right hand side represent transport due to diffusion, electric field gradient, and convection respectively. $K_{i,d}$ and $K_{i,c}$ represent the hindered nature of diffusion and convection of the ions inside the membrane.

The rejection of uncharged solutes for transport through a nanofiltration membrane has been modeled by continuum hydrodynamic model where porous membrane was considered as a bundle of straight cylindrical pores and solute transport is corrected for hindered convection and diffusion due to solute-membrane interactions [3,4]. Ion transport through NF membrane was initially modeled using irreversible thermodynamics. [5] However, the model failed to characterize the structural and electrical properties of the membrane because of lack of details of ion transport.

Electrokinetic space–charge model assumed the charged species as point charges and the transport of ions through pores is described as a creeping flow of these point charges through charged capillaries. The radial distribution of ions was defined by the Poisson Boltzmann equation while their transport through the pores was defined by extended Nernst–Planck equation mentioned above [6, 7]. However, these models have limited applications due to their numerical complexity in calculations [8].

One of the most widely adopted models of charged and uncharged solutes through NF membranes is Donnan-Steric Pore Model (DSPM) which takes into account the hindered transport, with equilibrium partitioning due to a combination of electrical (Donnan) and sieving (steric) mechanisms. This model is dependent on three parameters; effective pore radius, effective ratio of membrane thickness to porosity, and effective membrane charge density. It has been proven to be effective for monovalent ions and uncharged solutes. However, the model is not very accurate to model the transport of multivalent ions and mixture of electrolytes through NF membranes [1, 2]. A more recent approach, steric, electric and dielectric model (SEDE) considers the dielectric constant of solution along with the three parameters used in DSPM and reasonably predicts the transport and rejection of multivalent ions through nanofiltration membrane [9, 10].

Transport of water through Aquaporins has also been studied extensively [11-16]. Aquaporins are responsible for rapid transport of water through cell membrane. Aquaporin permeates water molecules across the cell membrane at a rate of 3×10^9 s^{-1} per channel. The functional unit of AqpZ consists of a tetramer. Each monomer of aquaporin provides an independent pore for water transport through cell membrane [17, 18]. Aquaporins have very high selectivity for water and they prevent the transport of solutes and ions from crossing the cell membrane. Protons might get a passage along with the water channels inside cell membranes by means of Grotthuss mechanism [19]. However, these protons get excluded in aquaporins with a very specific mechanism as a proton attempting to permeate aquaporin channel would have to work against the intrinsic bi-orientation of the water-file through an aquaporin pore [12, 16, 20].

3. Estimated PBI membrane diffusivity:

The concentration data for the unmodified PBI membrane in Table 1 converts to flux by calculating the change in concentration between the time steps assuming a constant flux for each time interval. The concentration across the membrane is also known allowing for calculation of diffusion coefficient by:

$$J = D \quad c \quad (2)$$

The mean of the calculated diffusivities across the time points represent the dotted and dashed black lines in Figure 11.

4. Diffusion studies:

In order to compare the salt diffusion rates through unmodified PBI and Aqp-SH modified PBI, the membrane was mounted in the middle of a stainless-steel diffusion cell to separate the two compartments (Salt solution side and DI water side). Each compartment in the cell was of volume 250 mL. The effective membrane area available for diffusion to occur was 3.5 cm^2. Continuous stirring was provided during the experiment. A solution of 1000 ppm NaCl solution was added into the salt solution side and the same amount of DI water solution was added into the other compartment. A sample of 2 mL was collected from each compartment every day. The experiment was run for 8 days to measure the rate of salt diffusion through the membranes. Salt concentrations of collected samples were measured using inductively coupled plasma (ICP) analysis. Initial conditions of the experimental setup is shown in Figure 2. Salt concentrations measured every day for all membranes are shown in Table 1.

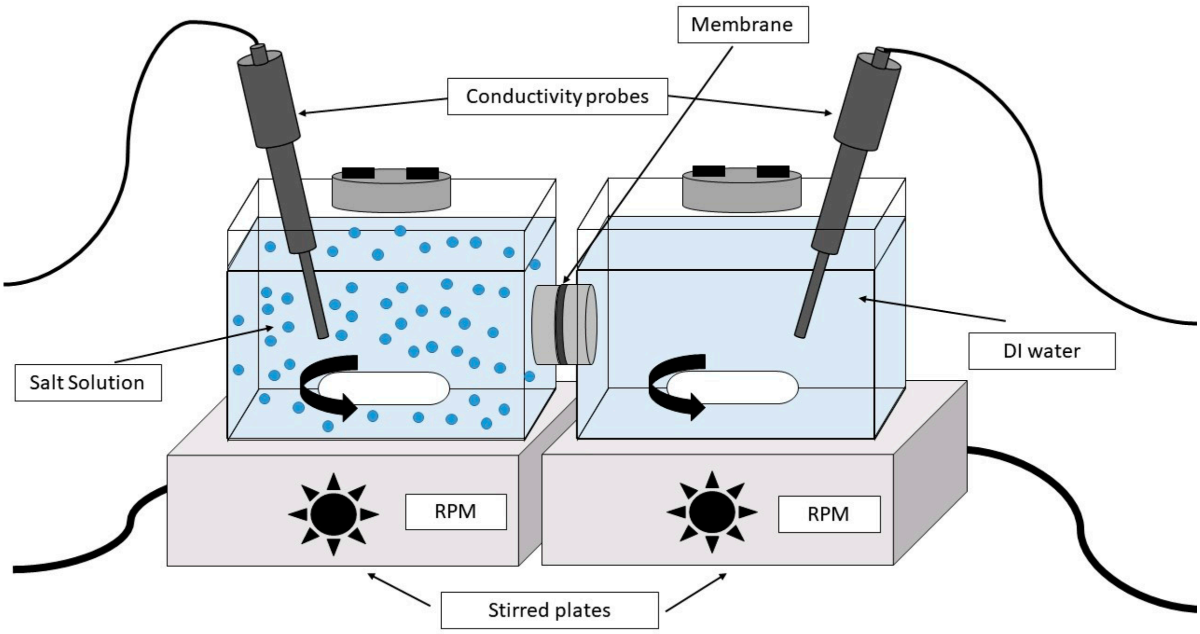

Figure S2. Diffusion cell assembly with 1000 ppm NaCl and DI water in two compartments separated by membrane.

Table S1. Salt concentrations measured every day for all three membranes in diffusion cell assembly.

	Unmodified PBI		PVA-alkyl modified PBI		Aqp-SH modified PBI	
	NaCl concentration (ppm)	H_20 concentration(ppm)	NaCl concentration (ppm)	H_20 concentration(ppm)	NaCl concentration (ppm)	H_20 concentration(ppm)
	NaCl concentration (ppm)	H_20 concentration(ppm)	NaCl concentration (ppm)	H_20 concentration (ppm)	NaCl concentration (ppm)	H_2O concentration(ppm)
Day 1	918.18	80.23	932.15	62.74	969.23	27.12
Day 2	829.35	167.2	851.35	142.75	922.46	72.34
Day 3	788.21	210.45	810.36	183.63	889.35	104.54
Day 4	743.1	253.73	770.12	225.76	872.53	124.65
Day 5	691.53	304.12	744.64	251.73	852.12	144.6
Day 6	676.16	318.63	725.91	270.82	837.81	160.32
Day 7	599.17	398.12	709.51	281.62	826.91	172.64

Day 8	597.81	399.65	705.23	287.13	815.63	179.5

List of symbols:

j_i Ion flux (based on membrane area) (mol $m^{-2}S^{-1}$)
$D_{i,p}$ Hindered diffusivity (m^2S^{-1})
C_i Concentration in membrane (mol m^{-3})
x Distance normal to membrane (m)
Z_i Valence of ion
R Gas constant ($Jmol^{-1}K^{-1}$)
T Absolute temperature (K)
F Faraday constant (C mol^{-1})
Ψ Electric potential in axial direction (v)
$K_{i,c}$ Hindrance factor for convection
V Solute velocity (ms^{-1})
